# Improved Vim targeting for focused ultrasound ablation treatment of essential tremor: A probabilistic and patient‐specific approach

**DOI:** 10.1002/hbm.25157

**Published:** 2020-08-06

**Authors:** Jason H. Su, Eun Young Choi, Thomas Tourdias, Manojkumar Saranathan, Casey H. Halpern, Jaimie M. Henderson, Kim Butts Pauly, Pejman Ghanouni, Brian K. Rutt

**Affiliations:** ^1^ Department of Radiology Stanford University Stanford California USA; ^2^ Department of Electrical Engineering Stanford University Stanford California USA; ^3^ Department of Neurosurgery Stanford University Stanford California USA; ^4^ Department of Neuroradiology Bordeaux University Hospital Bordeaux France; ^5^ INSERM U1215, Neurocentre Magendie University of Bordeaux Bordeaux France; ^6^ Department of Medical Imaging University of Arizona Tucson Arizona USA

**Keywords:** 7T MRI, essential tremor, MR guided focused ultrasound, surgical targeting, thalamus, Vim, white matter nulled MPRAGE

## Abstract

Magnetic resonance‐guided focused ultrasound (MRgFUS) ablation of the ventral intermediate (Vim) thalamic nucleus is an incisionless treatment for essential tremor (ET). The standard initial targeting method uses an approximate, atlas‐based stereotactic approach. We developed a new patient‐specific targeting method to identify an individual's Vim and the optimal MRgFUS target region therein for suppression of tremor. In this retrospective study of 14 ET patients treated with MRgFUS, we investigated the ability of WMnMPRAGE, a highly sensitive and robust sequence for imaging gray matter‐white matter contrast, to identify the Vim, FUS ablation, and a clinically efficacious region within the Vim in individual patients. We found that WMnMPRAGE can directly visualize the Vim in ET patients, segmenting this nucleus using manual or automated segmentation capabilities developed by our group. WMnMPRAGE also delineated the ablation's core and penumbra, and showed that all patients' ablation cores lay primarily within their Vim segmentations. We found no significant correlations between standard ablation features (e.g., ablation volume, Vim‐ablation overlap) and 1‐month post‐treatment clinical outcome. We then defined a group‐based probabilistic target, which was nonlinearly warped to individual brains; this target was located within the Vim for all patients. The overlaps between this target and patient ablation cores correlated significantly with 1‐month clinical outcome (*r* = −.57, *p* = .03), in contrast to the standard target (*r* = −.23, *p* = .44). We conclude that WMnMPRAGE is a highly sensitive sequence for segmenting Vim and ablation boundaries in individual patients, allowing us to find a novel tremor‐associated center within Vim and potentially improving MRgFUS treatment for ET.

## Graphical abstract

Magnetic resonance‐guided focused ultrasound (MRgFUS) ablation of the ventral intermediate (Vim) thalamic nucleus is an incisionless treatment for essential tremor (ET); a patient‐specific mapping of the upper extremity tremor‐associated center within Vim is an unmet need in present‐day MRgFUS treatment for ET. Using high quality preoperative and postoperative WMnMPRAGE imaging, state‐of‐the‐art nonlinear registration, segmentation of thalamic nuclei and FUS ablation characteristics, and probabilistic lesion calculations using a cohort of ET patients, we discovered a novel location within the Vim that is associated with upper extremity tremor reduction. This is a powerful example of human brain mapping that cannot be achieved in healthy subjects, yet still involves entirely noninvasive imaging and therapeutic modalities.

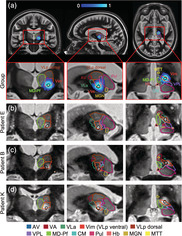

## 1 INTRODUCTION

Essential tremor (ET) is the most common movement disorder with a prevalence of approximately 4% in persons aged 40 and older. It is characterized by tremor during voluntary actions, most commonly in the hands, trunk, head, or voice. Medical management of ET typically relies on beta‐blockers or anti‐epileptic medications, but these drugs are ineffective in up to 50% of cases (Koller et al., 1994; Putzke et al., 2004). In these situations, intervention was previously limited to radiofrequency or radiosurgical thalamotomy, deep‐brain stimulation (DBS) (Benabid et al., 1996), or most recently, magnetic resonance‐guided focused ultrasound (MRgFUS) of the ventral intermediate (Vim) nucleus of the thalamus, although improvements tend to decrease over time for all methods. In the present study, we focused on MRgFUS thalamotomy, a promising new technology that was recently FDA‐approved as an incisionless ablation therapy for the relief of medically refractory ET (Elias et al., 2016; Halpern et al., 2019). Like DBS, preoperative imaging is used to determine an initial target location and awake intraoperative testing is used to refine the target location. For MRgFUS, intraoperative testing is done with iterative, reversible adjustments of the FUS focal spot location tracked with MR imaging, while monitoring for tremor reduction and adverse effects. Thus, MRgFUS allows real‐time feedback with modification of targeting and treatment parameters based on patient and imaging results. As an incisionless therapy, MRgFUS avoids the complications and risks associated with opening the skull. By comparison, radiosurgery, another incisionless therapy, uses a single target chosen before treatment, and the efficacy and side effects are not known for several months after treatment. However, unlike DBS, in which the stimulation treatment can be adjusted or even reversed after the implantation operation, FUS ablation makes a permanent lesion; this places an even higher emphasis on targeting accuracy.

The current standard method of identifying the Vim during presurgical planning uses stereotactic atlas coordinates derived from the Vim in the Tailarach brain with limited individual‐specific adjustment based on the proportional distance between the anterior commissure (AC) and posterior commissure (PC) and the distance from the ventricular wall (Børretzen et al., 2014; Obwegeser et al., 2001). This targeting is typically accomplished with pretreatment T2‐weighted MR and computed tomography (CT) imaging. These coordinates then serve as the initial preoperative target that is intraoperatively verified and adjusted if necessary using awake testing for treatment‐induced reductions in tremor and adverse effects and, in the case of DBS, recordings of the known electrophysiological signature of the Vim. While many ET cases have been treated using this standard targeting method with good clinical outcomes across these treatment options (Ravikumar et al., 2017), there are significant individual differences in brain anatomy, which can contribute to targeting error. These include variable distances between the PC and thalamic nuclei (Brierley & Beck, 1959) and between stereotactically derived Vim and its connected and adjacent fiber tracts (Anthofer et al., 2014). Accurate initial targeting is important for patient outcome and efficiency of the MRgFUS procedure. Specifically, while FUS intraoperative focal spot mapping can be accomplished, allowing iterative refinement of the treatment location with reversible low‐energy tissue sonications, the cumulative effect of many such sonications can result in nonspecific, gradual heating and edema that can obscure treatment‐specific effects and contribute to adverse events. Thus, a targeting method that identifies a patient's own Vim and an optimal target therein could improve patient outcome and efficiency of the MRgFUS procedure.

Recent work has been aimed at a personalized, albeit indirect, identification of the Vim using diffusion tensor imaging (DTI) and known anatomical connectivity; however, there is still a lack of consensus on the best tracts to model (Akram, Hariz, & Zrinzo, 2019; Middlebrooks, Grewal, & Holanda, 2019). One promising approach is targeting based on directly visualizing the Vim, which would allow the Vim to be identified based on a patient's own brain anatomy. Standard T1‐ and T2‐weighted MRI protocols have not provided sufficient contrast to directly visualize the Vim in an individual patient. However, several studies have investigated nonstandard sequences for direct visualization of the Vim. These include FGATIR and WAIR sequences that null the white matter signal (Sudhyadhom, Haq, Foote, Okun, & Bova, 2009; Vassal et al., 2012), as well as susceptibility‐weighted imaging (Abosch, Yacoub, Ugurbil, & Harel, 2010; Deistung et al., 2013) and proton density mapping (Spiegelmann, Nissim, Daniels, Ocherashvilli, & Mardor, 2006). Thus far, none of these sequences have become standard procedure for MRgFUS, although FGATIR is gaining common usage and there is ongoing work investigating the application of several of these sequences for novel DBS targeting methods (Hemm et al., 2016; Morishita et al., 2019). Patient‐specific mapping of the upper extremity tremor‐associated center within Vim is therefore an unmet need in present‐day MRgFUS treatment for ET.

**FIGURE 1:**
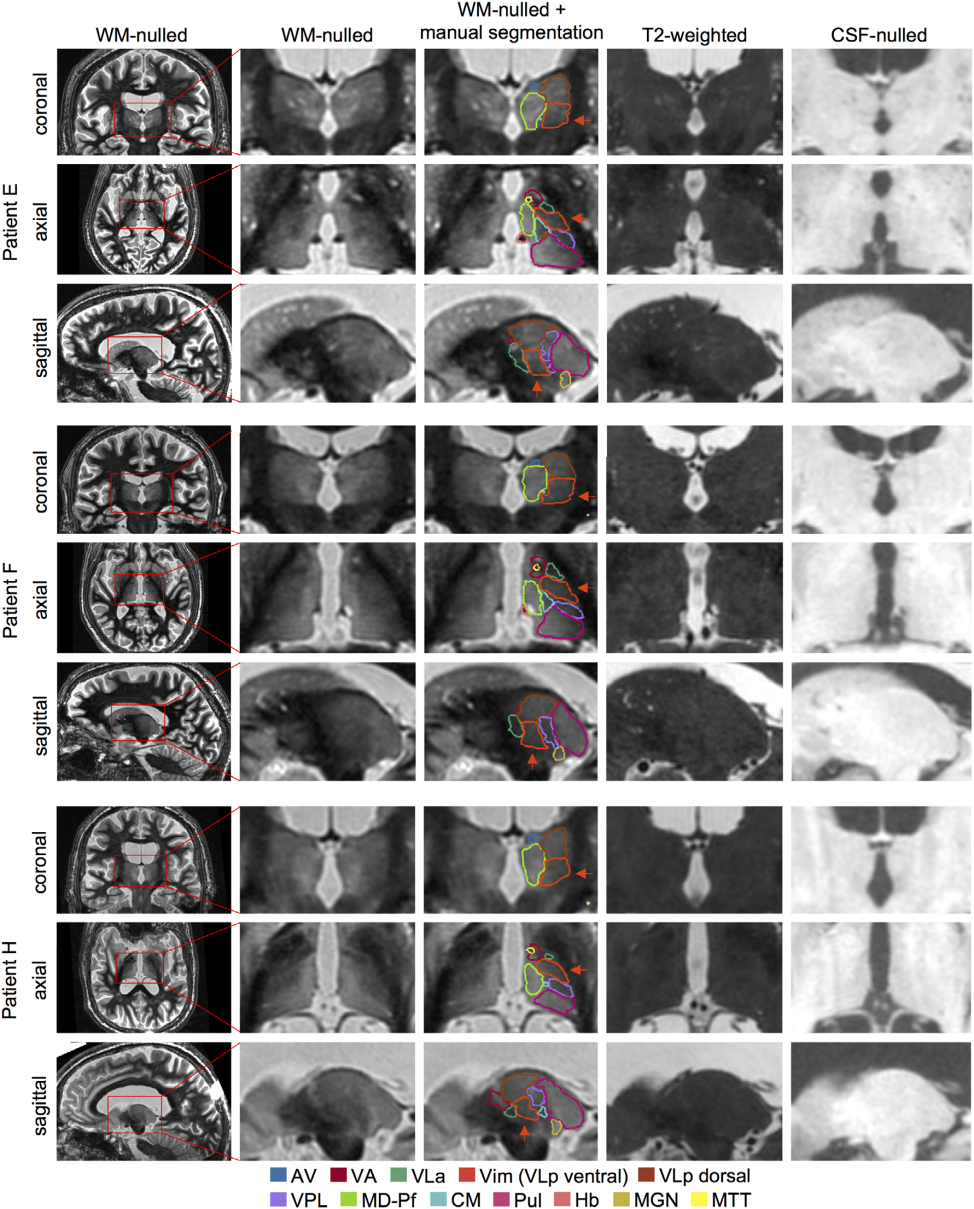
Comparison of WMnMPRAGE images with standard T2‐weighted and CSFnMPRAGE images for three representative patients. Coronal, axial, and sagittal slices are shown centered on the Vim nucleus. The greater sensitivity of WMnMPRAGE imaging provides higher contrast between thalamic nuclei than the standard imaging protocols, which allows manual and automated segmentations of these nuclei. Note: Due to the cytoarchitectonically gradual progression between the Vim and the more dorsal part of VLp, the VLp was manually segmented and the Vim (orange arrow) was defined as the ventral half of the VLp. AV, anteroventral; CM, centromedian; Hb, habenula; MD, mediodorsal; MGN, medial geniculate nucleus; MTT, mammillothalamic tract; Pf, parafascicular; Pul, pulvinar; VA, ventral anterior; VLa, ventral lateral anterior; Vim, ventral intermediate; VLp, ventral lateral posterior; VPL, ventral posterior lateral

In this work, we developed new methodology to find a location within Vim that is associated with upper extremity tremor, with the aim of improving MRgFUS treatment for ET. We began by investigating the ability of the white‐matter‐nulled magnetization prepared rapid gradient echo (WMnMPRAGE) sequence (Bluestein et al., 2012; Saranathan, Tourdias, Bayram, Ghanouni, & Rutt, 2015; Tourdias, Saranathan, Levesque, Su, & Rutt, 2014), a highly sensitive, robust, and reliable sequence that achieves high gray matter‐white matter contrast, to directly visualize and segment both the Vim and the FUS ablation. Like FGATIR and WAIR, WMnMPRAGE has a shorter inversion time than the standard cerebrospinal fluid‐nulled MPRAGE (CSFnMPRAGE), leading to dark white matter and bright CSF, as well as improved distinction between subcortical gray matter structures like the nuclei of the thalamus compared to standard T2‐weighted or T1‐weighted CSFnMPRAGE images (Figure 1). WMnMPRAGE is different from FGATIR and WAIR in that it uses different sequence parameters and k‐space trajectory, providing robustness to motion, as well as being optimized for SNR efficiency and intrathalamic contrast (Saranathan et al., 2015). We have previously shown that WMnMPRAGE is not only highly sensitive to normal thalamic substructures, but also to multiple sclerosis lesions (Planche et al., 2019); this motivated our use of this sequence for visualizing and segmenting the FUS ablation zones in the present study.

Previously, we found that the mammillothalamic tract and 11 thalamic nuclei, including the ventral lateral posterior (VLp) nucleus of which Vim is the ventral portion (Mai & Majtanik, 2019), could be visually identified and manually segmented from WMnMPRAGE images in healthy subjects and multiple sclerosis patients (Tourdias et al., 2014). Subsequently, based on 20 manually segmented subjects, we trained a multi‐atlas automated segmentation method named THalamus Optimized Multi Atlas Segmentation (THOMAS) to accurately identify and segment these 12 thalamic structures in WMnMPRAGE images (Su et al., 2019). THOMAS segmented these 12 thalamic structures with a high degree of accuracy (Dice = 0.70–0.85; volume similarity index [VSI] = 0.82–0.97; metrics described by Dice [1945] and Taha and Hanbury [2015]) in an independent test set of 8 manually segmented subjects (Su et al., 2019). Recent work has used THOMAS to characterize abnormal thalamic nuclei volumes and investigate their relation to disease symptoms in multiple sclerosis, HIV, and alcoholism (Planche et al., 2019; Zahr, Sullivan, Pohl, Pfefferbaum, & Saranathan, 2019). Due to its fast, unbiased, and automated nature, THOMAS is a promising method for efficiently identifying thalamic nuclei, including the Vim, for clinical use.

We then applied these sequence and segmentation methodologies to a retrospective study of MRgFUS‐treated patients. We investigated (a) the ability of WMnMPRAGE to visualize and identify the Vim in individual ET patients with manual and THOMAS automated segmentations, (b) the ability of WMnMPRAGE to identify relevant FUS ablation zones, and (c) the usage of this information in a probabilistic analysis to find a precise target region within the Vim for initial targeting that best correlates with clinical outcome. We hypothesized that WMnMPRAGE imaging would allow us to outline the Vim directly within individual patients both manually and using THOMAS, as well as the extent of the FUS ablation. We further hypothesized that, using this enhanced location information, we could identify a critical target region of ablation within the Vim that would be a better predictor of tremor reduction than the current standard, stereotactic initial targeting method based on anatomical landmarks and Talairach coordinates.

## 2 METHODS

### 2.1 Overview

We obtained WMnMPRAGE images from 14 ET patients who underwent MRgFUS treatment. We first identified each patient's Vim through manual segmentation and the THOMAS automated segmentation using preoperative WMnMPRAGE images. The accuracy of the THOMAS segmentation of the Vim and other thalamic nuclei was assessed by Dice and VSI overlap measures in comparison to the manual segmentations. We next examined postoperative WMnMPRAGE images to detect the boundaries of the FUS ablation using a semi‐automated ablation segmentation method, and compared ablation volumes and locations for each patient with his or her own Vim boundaries. We next examined how different ablation characteristics correlated with clinical outcome at 1 month across the patients with an approach similar to probabilistic lesion mapping (Atkinson et al., 2002) or voxel‐based lesion‐symptom mapping (Bates et al., 2003). In short, we nonlinearly warped each patient's brain into a group‐specific, average brain to allow direct comparison of these Vim and ablation metrics across all patients. We also obtained a probabilistic target region defined as the volume of overlap between the ablations across all patients, further eroded to 20 mm^3^, which approximated the roughly spherical average volume contained by the boundary of the ablation core. This volume includes both hypointense and hyperintense core regions, likely corresponding to the Zones I and II of the ablation still present at 1 month described by Wintermark et al. (2014); we will refer to this volume as the ablation core volume for brevity. We investigated whether a proximity‐to‐target metric, defined as the overlap of each patient's ablation core with this probabilistic target region, correlated with clinical outcome at 1 month. The 1‐month timepoint was selected for assessing clinical outcome based on the availability of a complete set of clinical outcome measurements for all patients, as well as being a timepoint that is both sufficiently close in time to the MRgFUS procedure to show strong therapeutic effects, but sufficiently delayed to allow for decreased edema and reduced adverse events (Chang et al., 2015; Harary, Essayed, Valdes, McDannold, & Cosgrove, 2018; Jung et al., 2015). Lastly, we tested to see if this proximity‐to‐probabilistic‐target metric was a better predictor of clinical outcome than the corresponding proximity metric computed between the ablation core and the standard initial target.

### 2.2 Subjects

Fourteen consecutive patients (7 males, 11 right‐handed, mean age 75.5 years, age range 69–86 years at the time of the preoperative MRI scans) with severe ET that was refractory to medication were enrolled for MRgFUS treatment at the Stanford Movement Disorders Clinic between August 2013 and April 2016. Throughout this article, we refer to these patients by anonymized alphabetic codes A‐N. Informed consent was obtained from all patients, who were part of a larger prospective, multicenter study (Elias et al., 2016). The research was approved by the Institutional Review Board of Stanford University.

### 2.3 Scanning protocols

Patients were imaged prior to treatment on a GE Discovery MR950 7T MRI scanner (GE Healthcare) using a 32‐channel head coil (Nova Medical). In addition to conventional localizers and anatomical scans, a WMnMPRAGE scan was acquired for thalamic visualization and segmentation. This sequence used the following scan parameters: inversion repetition time (TS): 6000 ms, inversion time (TI): 680 ms, excitation repetition time (TR): 10.1 ms, echo time (TE): 4.6 ms, bandwidth (BW): ±12 kHz, flip angle (FA): 4°, field‐of‐view (FOV): 18 cm, slice thickness: 1 mm, slice number: 220, matrix: 180 × 180, k‐space ordering; 2D radial fanbeam, ARC parallel imaging acceleration: 1.1 × 1.1, scan time: 10.1 min (Saranathan et al., 2015). In one case, the 7T images could not be acquired due to a hardware failure (Patient C); for this subject we used the corresponding 3T WMnMPRAGE images (scan time: 10.2 min).

Pretreatment and post‐treatment 3T MR images were acquired on a GE Discovery MR750 3T MRI scanner (GE Healthcare) using an 8‐channel head coil (In Vivo). Coronal WMnMPRAGE images were acquired with the following parameters: TS: 4500 s, TI: 500 ms, TR: 10.4 ms, TE: 4.6 ms, BW: ±12 kHz, FA: 7°, FOV: 18 cm, slice thickness: 1 mm, matrix: 180 × 180 × 200, k‐space ordering: 2D radial fanbeam, no parallel imaging, scan time: 10.2 min with prospective motion correction (Saranathan et al., 2015).

The 7T and 3T WMnMPRAGE pretreatment scans were collected several weeks prior to treatment. The 3T WMnMPRAGE immediate post‐treatment images were acquired after removing the FUS transducer and head frame and repositioning the patient within the 8‐channel head coil. The study protocol did not include WMnMPRAGE imaging at later timepoints (i.e., a few weeks or months post‐FUS, for example after edema has resolved) The WMnMPRAGE images obtained as described above were used for the retrospective analyses described in this article and not for any form of prospective preoperative planning.

### 2.4 MRgFUS treatment

1–4 weeks after the pretreatment scans, patients received unilateral MRgFUS treatment (ExAblate 4,000, InSightec, Israel) of the Vim nucleus. A stereotactic frame was placed on the patient with the FUS transducer. The patient was then positioned in the 3T MRI scanner for MRgFUS. FRFSE and FIESTA‐C images were acquired to locate the AC and PC landmarks. The initial target for the ablation center point was defined from a Talairach‐based target on the AC‐PC plane at the inferior boundary of the Vim, on the side contralateral to the hand being targeted for tremor reduction. This target point was located 11 mm lateral to the wall of the third ventricle, 0.25 × AC‐PC length anterior to the PC, and at the AC‐PC plane. The FUS focal spot was visualized and verified with respect to this initial target using low energy sonications and MR thermometry in three orthogonal planes. Treatment proceeded by increasing sonication energy until a temperature of 45–50°C was achieved at the focus, as assessed by MR thermometry. The patient was then moved out of the scanner and the tremor reduction in the upper extremity contralateral to the lesion assessed. If the reduction was poor, or if there were side effects, the ablation target region was adjusted or enlarged within the AC‐PC plane. This iterative cycle of adjustment, temperature scan, and tremor test was repeated until a satisfactory location was found that reduced tremor, ideally without causing side effects. High‐energy sonication was then applied at this location until a tissue temperature of 55–60°C, guaranteeing tissue ablation. Following this ablation, the treatment outcome was reassessed. If clinically necessary, sonication continued, either at the current target to grow the lesion, or after adjusting the focus to address residual symptoms. At the completion of the treatment, the frame was removed, and the patient was transferred from the FUS table to a gurney and returned to the 3T scanner to acquire high quality post‐treatment WMnMPRAGE images using an 8‐channel brain coil.

Patients were ablated in the contralateral thalamus corresponding to the dominant hand: three patients were ablated in the right thalamus (Patients B, D, and L), while 11 patients were ablated in the left thalamus. In order to properly compare these right hemisphere‐ablated patients to the rest of the cohort, their images were reversed right to left (using the FSL *fslswapdim* command) for all registrations and analyses. This procedure guaranteed that the ablation zones were always on the left side of the template space.

### 2.5 Clinical outcome measure

We evaluated therapeutic improvements using the Clinical Rating Scale for Tremor (CRST) A + B (Stacy et al., 2007), the treated hand tremor subscore of the CRST, administered immediately before treatment and at 1 month post‐treatment (Table 1). This tremor subscore with a maximum of 32 points was calculated by summing the observed and the performance‐based scores from parts A and B for the treated hand (Elias et al., 2016; Wintermark et al., 2014). Tremor assessments were performed by neurologists specialized in movement disorders. Following FUS ablation, patients showed significant reduction in dominant hand tremor at the 1‐month timepoint compared to pretreatment baseline (*t* = 9.74, *p* = 2.4 × 10^−7^, Table 1).

**TABLE 1:** CRST A + B clinical outcome scores. Patient outcomes at baseline and 1 month for dominant hand tremor (contralateral Vim was targeted by MRgFUS) as measured by CRST A + B

	CRST A + B	CRST A + B
Patient	Baseline	1 month
A	21	15
B	25	12
C	11	4
D	20	5
E	20	2
F	20	8
G	21	5
H	22	19
I	18	4
J	23	8
K	22	7
L	25	10
M	19	8
N	14	8
Mean ± *SD*	20 ± 3.78	8.21 ± 4.61

*Note:* Dominant hand tremor significantly improved at 1 month compared to baseline (*t* = 9.74, *p* = 2.4 × 10^−7^).

### 2.6 Postprocessing overview

**FIGURE 2:**
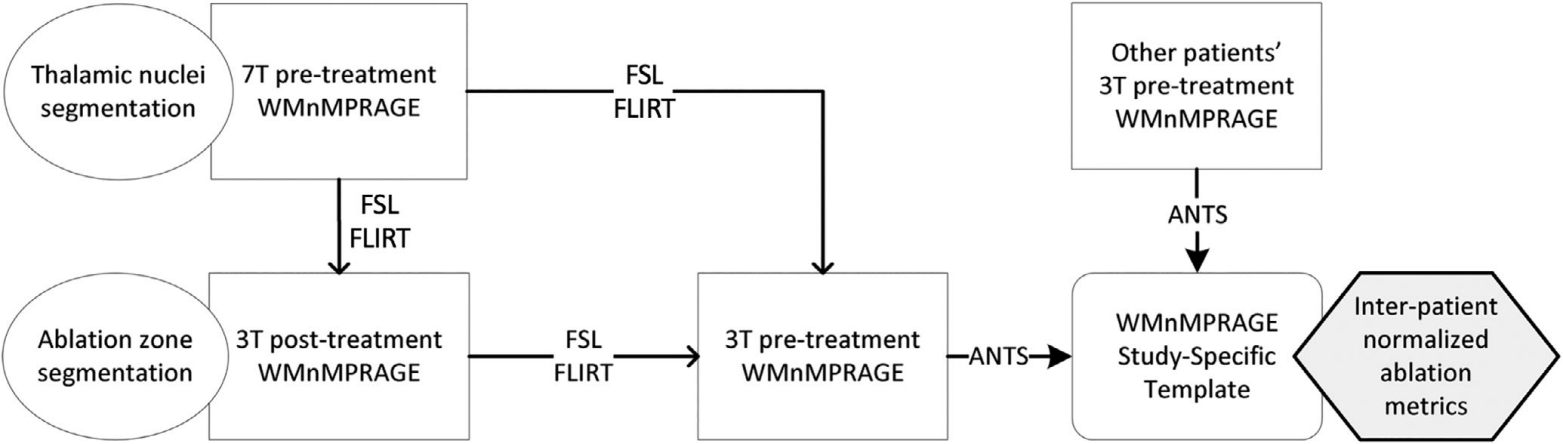
Overview of preprocessing and registration pipeline. Generally, 7T pretreatment WMnMPRAGE images were used for thalamic segmentations; these were linearly registered to the 3T pretreatment WMnMPRAGE volume for each patient, and nonlinear warps were applied to transform them into the group normalized brain template space. 3T post‐treatment images were used to detect and characterize ablation features; these were first linearly registered to the 3T pretreatment WMnMPRAGE volumes and then nonlinearly transformed to the group normalized brain template space. The study‐specific group normalized brain template was created from all subjects' 3T pretreatment WMnMPRAGE images. With all relevant image volumes transformed to the template space, and relevant features of the initial targets and final ablations derived in the template space, we obtained interpatient ablation metrics and conducted correlation analyses with clinical outcome across patients. See Section 2 for further details

Figure 2 shows an overview of the postprocessing pipeline. In brief, the 3T pretreatment WMnMPRAGE imaging was used to generate a study‐specific, group normalized brain template by registering and averaging the images from all subjects. Within this template space, metrics such as the boundaries, centers of mass, and volumes of the ablation and Vim regions were measured. The 7T pretreatment WMnMPRAGE images were used for thalamic segmentations (except for Patient C, for whom the 3T pretreatment WMnMPRAGE image was used); these were linearly registered to the 3T pretreatment WMnMPRAGE volume for each subject (except for Patient A, for whom the 7T pretreatment WMnMPRAGE image was used, due to severe motion artifacts in the 3T pretreatment WMnMPRAGE), and nonlinear warps applied to transform them into the normalized brain template space. The 3T post‐treatment images were used to detect and characterize ablation features; these were first linearly registered to the 3T pretreatment WMnMPRAGE volumes and then nonlinearly transformed to the normalized brain template space. All linear and nonlinear transformations were checked by eye for accuracy. With all relevant image volumes transformed to the template space, and relevant features of the initial targets and final ablations derived in the template space, we conducted group analyses to identify metrics associated with improved clinical outcome.

#### 2.6.1 Intrasubject co‐registration

All images were first corrected with the N4BiasFieldCorrection algorithm within the Insight Segmentation and Registration Toolkit (ITK) (Tustison et al., 2010). Linear registration was used to transfer segmented regions, such as the thalamic nuclei or ablation zones, between images acquired on the same patient but at different field strengths or timepoints.

Pretreatment 3T and 7T WMnMPRAGE images were aligned to post‐treatment 3T WMnMPRAGE using FSL's FLIRT (FMRIB's Linear Image Registration Tool) with 9° of freedom and a binary weighting mask applied to the post‐treatment reference image (Jenkinson, Bannister, Brady, & Smith, 2002). These options visibly improved the registration quality. A transform with 9° of freedom allowed scaling in all dimensions. This helped account for some of the variation in image sizes and shapes between field strengths. The use of a binary weighting mask allowed us to selectively ignore parts of the image that could harm the registration accuracy, such as image artifacts and the ablation hyperintensity in the post‐treatment image. The mask was generated with FSL's BET (Brain Extraction Tool) on the post‐treatment 3T WMnMPRAGE image (Smith, 2002). The resulting mask contained a partial segmentation of the whole brain, since BET was not designed for the white‐matter‐nulled contrast. However, the resulting mask successfully excluded the tissues outside of the brain and brain regions near sinuses that were corrupted by artifacts from field inhomogeneities, and was sufficient for the purpose of weighting the registration cost‐function. The mask was further modified by excluding the ablation zone, as defined by the manual segmentation described above, and by performing a binary erosion with a 7 × 7 × 7 cubic kernel to the whole mask to make it more conservative.

The 7T pretreatment WMnMPRAGE volume was aligned to the 3T pretreatment WMnMPRAGE volume in a comparable fashion. First, N4BiasFieldCorrection, then BET on the 3T‐pre reference image with a 7 × 7 × 7 cubic binary erosion, and, finally, FLIRT with 9° of freedom and the eroded reference brain mask. Removing the ablation region was not necessary here since it did not exist on the 3T pretreatment images.

#### 2.6.2 Intersubject registration

**FIGURE 3:**
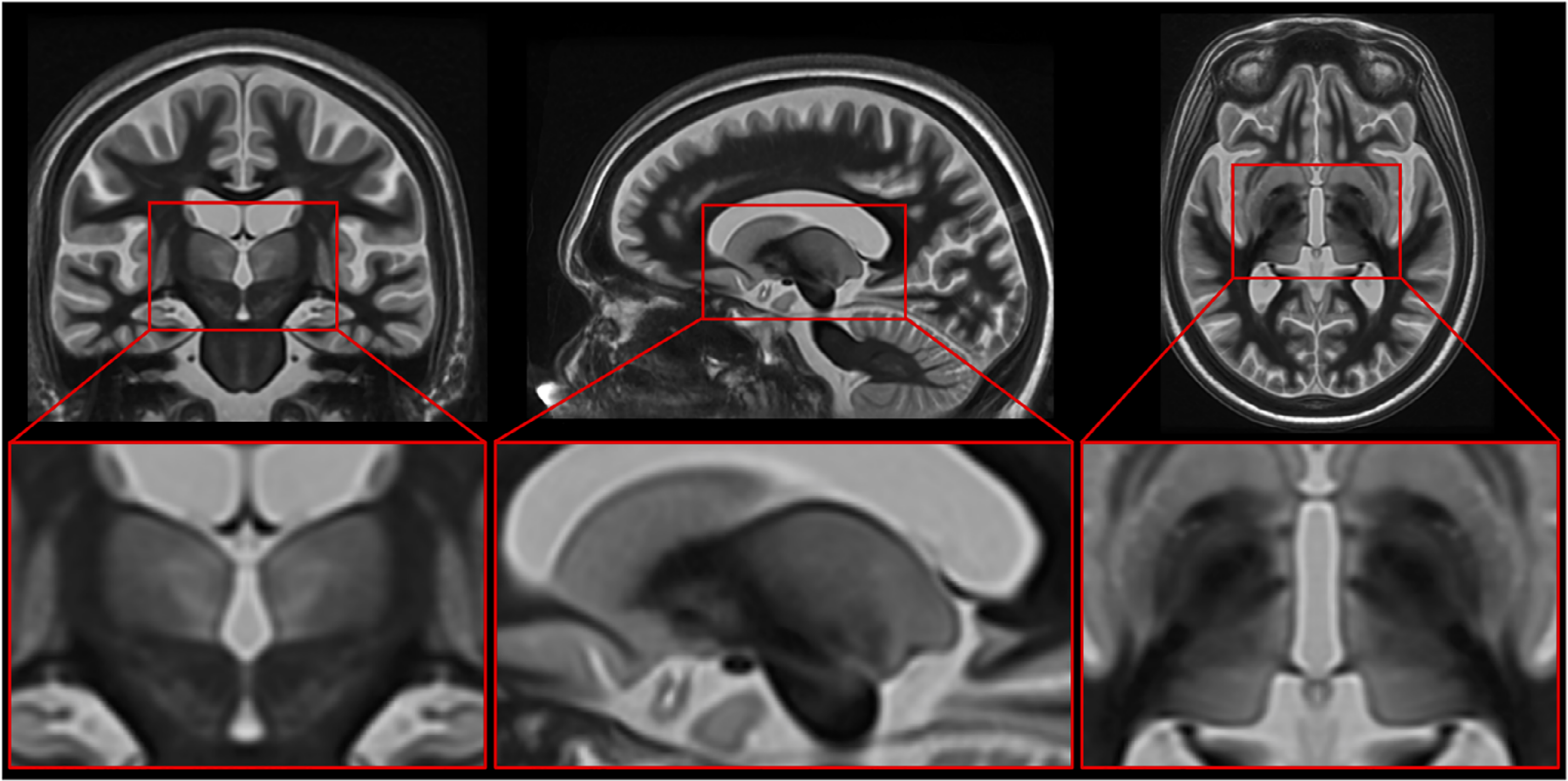
Study‐specific, group normalized brain template. A study‐specific template was created to enable direct comparison of the Vim and ablation regions across patients. The template was created from the 3T pretreatment WMnMPRAGE scans of 13 patients (scans flipped right to left if ablations were made in the right hemisphere). Image volumes were first nonlinearly registered using ANTs and then averaged to form the normalized average brain. See Section 2 for further details

A study‐specific template (or “average brain”) (Figure 3) was created to enable direct comparison of the ablation regions between subjects. This approach of building a study‐specific template is preferred over the use of a single reference subject for registration purposes because it does not bias the brain coordinate space toward any one particular subject (Avants et al., 2010). The study‐specific template was created from the 3T pretreatment WMnMPRAGE scans of 13 patients out of the 14 total patients. Patient A's scan suffered from motion artifacts and was therefore excluded from the template creation. For this subject, the 7T pretreatment WMnMPRAGE image was afterwards nonlinearly registered to the template using ANTs (Advanced Normalization Tools) but was not used in the formation of the template. For template formation, the 3T pretreatment WMnMPRAGE image volumes were first nonlinearly registered using ANTs (*buildtemplateparallel.sh*) and then averaged to form the study‐specific template (Avants et al., 2010; Avants, Epstein, Grossman, & Gee, 2008). Since subjects were not all treated on the same side of the brain, and we wanted to make direct comparisons between left‐ and right‐treated patients in the same normalized template space, we developed a strategy to symmetrize the template in the region of the thalamus, by feeding both the original images and their left–right mirrored copies into the template building algorithm. The template building algorithm within ANTs (*buildtemplateparallel.sh*) was set by default to perform 4 iterations for the template generation process, but 16 iterations were used in this study to ensure excellent convergence. The result of these operations was a normalized average brain, which was highly symmetric in the region of the thalamus, and the corresponding nonlinear transformation matrix from each of the subjects to this space.

To confirm the accuracy of the nonlinear warping, we registered all 14 patients to the group template, and then evaluated the Dice overlap between each patient's segmented nuclei and the corresponding nuclei for every other patient. The median Dice overlap was 0.711 for VLP (which includes Vim) and 0.886 for the whole thalamus. This shows very good registration accuracy, surpassing the median overlap scores from Klein et al. (2009) testing accuracy across a range of brain structures and algorithms. We also assessed the effects of nonlinear warping by quantifying the distances between standard initial targets that were linearly or nonlinearly warped to the patient space and anatomical landmarks used to derive the standard initial target (i.e., AC, PC, the percent of the AC‐PC distance from the PC, and the AC‐PC plane), as well as the Euclidean distance between these linearly and nonlinearly warped initial targets (Table 2). We found that the Euclidean displacement between the linearly versus nonlinearly warped targets in patient space ranged up to 2.4 mm (Patient B), and that this displacement was individual‐specific and not consistent across patients.

**TABLE 2:** Distances in mm from the standard initial target linearly or nonlinearly warped to patient space, to various anatomical landmarks: midline, posterior commissure (PC), the percent of the anterior commissure (AC)‐PC distance from the PC, and the AC‐PC plane

	Distance (mm) from standard initial target linearly warped to patient space, to various landmarks				Distance (mm) from standard initial target nonlinearly warped to patient space, to various landmarks				Euclidean distance (mm) between standard initial targets warped linearly and nonlinearly to patient space
Patient	To midline along *X*‐axis	To PC along *Y*‐axis	% AC‐PC distance	To AC‐PC plane along *Z*‐axis	To midline along *X*‐axis	To PC along *Y*‐axis	% AC‐PC distance	To AC‐PC plane along *Z*‐axis	Increment between targets
A	13.2	7.2	25.2	−0.1	13.3	5.8	20.2	0.6	1.6
B	14.7	6.7	25.8	0.5	12.5	7.3	28.2	1.1	2.4
C	13.0	8.1	27.3	−1.2	13.6	7.3	24.8	−1.1	1.0
D	16.3	6.5	25.0	−0.8	15.3	6.4	24.4	−0.4	1.1
E	13.1	5.7	22.7	−0.8	13.8	5.9	23.4	0.3	1.4
F	13.1	6.9	24.5	0.5	14.1	5.3	19.0	0.2	1.9
G	13.7	7.0	25.1	0.5	13.4	5.9	21.2	−0.4	1.4
H	13.2	6.5	24.5	0.9	13.1	6.6	25.0	1.3	0.4
I	13.3	6.6	23.5	−0.2	14.5	6.2	21.9	−0.8	1.5
J	12.6	6.7	25.0	0.6	13.8	6.0	22.3	−0.7	1.9
K	11.7	6.7	25.9	0.2	12.7	6.3	24.4	−0.3	1.2
L	13.9	7.0	27.3	0.3	12.9	6.1	24.0	0.6	1.4
M	13.2	6.7	25.1	−0.3	13.3	7.0	26.6	0.2	0.6
N	13.9	6.2	24.6	0.4	12.8	5.6	22.5	0.4	1.2
**Mean**	**13.5**	**6.7**	**25.1**	**0.0**	**13.5**	**6.3**	**23.4**	**0.1**	**1.4**
***SD***	**1.1**	**0.5**	**1.3**	**0.6**	**0.8**	**0.6**	**2.5**	**0.7**	**0.5**

*Note:* Last column shows Euclidean distance increment between standard initial target coordinates, linearly versus nonlinearly warped to patient space.

Figure 3 depicts the study‐specific template centered on the Vim. The template has increased contrast within the thalamus compared to the contrast seen in WMnMPRAGE images for individual brains, allowing for improved distinction between individual nuclei. There is also a high degree of right–left symmetry that is apparent for subcortical brain structures including the thalamus but breaks down for some cortical structures. Since these cortical structures were not relevant to the study, we believe the template converged to a solution sufficient for intersubject comparison of left and right thalamic structures. The group‐level thalamic segmentation shown in Figure 6 overlaid on this template was created from the manual segmentations of all patients using a majority‐vote method for each voxel.

The post‐treatment space containing the ablation was then aligned to this normalized space by concatenating the linear and nonlinear transformations from 3T post‐treatment to 3T pretreatment to the template (see the overall registration strategy in Figure 2). Patient A's 3T pretreatment image was of poor quality, so transformations were instead chained from 3T post‐treatment space to 7T pretreatment space to the template. Direct registration of the 3T post‐treatment images to the template would have had poor accuracy in the vicinity of the bright ablation zone, which is exactly the critical region we sought to evaluate. To remedy this, a simple global linear transform was used to align the post‐treatment images to the pretreatment images, while masking out the ablation zone from the registration and nonlinearly registering the pretreatment WMnMPRAGE images to the group template. This enabled accurate registration of the post‐treatment thalamus to the template, unaffected by the presence of the ablation lesion.

### 2.7 Ablation and thalamic segmentations

#### 2.7.1 Ablation core and penumbra semi‐automatic segmentation

The outer boundary of the entire hyperintense region (i.e., the border between the penumbra and normal tissue) was first manually traced using 3D Slicer (Fedorov et al., 2012). The ablation core was then defined within this region using an automated algorithm as follows. The median and 99th percentile image intensity values were found within the normal‐appearing thalamus. The image intensity values (*x*) in the ablation region were then re‐scaled with 0 at the median and 1 at the 99th percentile of normal‐appearing thalamus: (*x* – median)/99th percentile. The 99th percentile was used instead of the absolute maximum in case the whole thalamus segmentation erroneously included a small portion of the ventricles, which appear strongly hyperintense. Voxels with scaled intensity values greater than 1.25 were defined as the core. This value was chosen empirically based on visual comparison to manual segmentations of the core in four cases. This result was post‐processed with a binary closing operation provided by the scikit‐image Python library using a connectivity‐1 kernel (a 3D cross or jack) to fill in the hypointensity found within some patients' cores.

#### 2.7.2 Thalamic nuclei manual segmentation

For each patient except Patient C, 12 thalamic structures and the whole thalamus were manually segmented from the 7T pretreatment WMnMPRAGE images (which provided better thalamic contrast than the 3T pretreatment WMnMPRAGE images) by an expert neuroradiologist (TT, 10 years of experience) with guidance from the Morel atlas, using a previously validated method (Morel, Magnin, & Jeanmonod, 1997; Tourdias et al., 2014). For Patient C whose 7T images could not be acquired, the 3T pretreatment WMnMPRAGE images were instead used for manual segmentation. All segmentations were performed while blinded to the post‐treatment MRI scan. The Vim nucleus of the Schaltenbrand and Wahren atlas (Schaltenbrand, Spuler, Wahren, & Rümler, 1971) corresponds to the ventral portion of the VLp nucleus of the Morel atlas, abutting the inferior border of VLp (Mai & Majtanik, 2019). While the overall VLp body can be delineated using WMnMPRAGE imaging (Tourdias et al., 2014), there is a dorsal‐ventral cytoarchitectonic gradient, rather than a sharp internal boundary, between the Vim and the dorsal portion of VLp (Morel et al., 1997). For this reason, the Vim was approximated in each patient as the ventral half of VLp, defined by splitting the manually segmented VLp into dorsal and ventral halves using a transaxial cutting plane midway between the two transaxial planes that bound VLp.

#### 2.7.3 Thalamic nuclei automated segmentation

THOMAS was run as described previously (Su et al., 2019) on the noncropped 7T WMnMPRAGE images for each patient (except Patient C, for whom 3T WMnMPRAGE images were used) to obtain automated segmentations of the thalamic nuclei. The THOMAS‐derived Vim was then defined as the ventral half of the VLp output of the THOMAS segmentation. Dice and VSI (Dice, 1945; Taha & Hanbury, 2015) values were calculated using the manual segmentations created from the same images, as described above.

### 2.8 Clinically efficacious target analysis

#### 2.8.1 Ablation metrics

With the manually segmented thalamic anatomy and ablation zone segmentations aligned to the post‐treatment images, Figure 5 was created showing ablation locations within the thalamus. These segmentations were transformed to the study‐specific template, enabling distance and volume measures to be normalized and compared between subjects, which were used to evaluate how the location and extent of the ablation correlated with clinical outcome at 1 month. Note that each patient's own Vim, derived from manual segmentation warped to the template space, was used for these metrics. This removed any error associated with atlas‐derived Vim characteristics. We considered the following individual patient‐level metrics: the volume of the whole ablation or core alone, the fraction of the Vim volume covered by the whole ablation or core alone, the distance between the ablation core and the Vim's centers of mass, and the distance between the ablation core's center of mass and the standard initial target coordinate.

#### 2.8.2 Probabilistic target region

In addition to these individual patient‐level metrics, we defined a new imaging‐derived target region comprised of the intersection of all patient ablation zones. The hypothesis was that since all subjects consistently experienced at least some clinical improvement immediately following treatment, the region of the thalamus that was most consistently affected by FUS across all subjects as seen in the immediate post‐treatment imaging would correspond to the optimal treatment target. Within the normalized template space, the intersection of all patients' whole ablation zones was computed and eroded by a 3 × 3 × 3 voxel kernel down to a volume of 20 mm^3^, corresponding to the average across all patients' ablation core volumes. The fraction of this probabilistic target region covered by each patient's ablation core was quantified and plotted against 1‐month clinical outcome scores.

To compare the performance of this probabilistic region with the standard initial target region defined by the standard stereotactic targeting method, we decided to perform this analysis in each patient's native space, simulating new patient cases. We first defined coordinates using the standard targeting method in the 3T pretreatment WMnMPRAGE space (except for Patient A, for whom it was defined in the 7T pretreatment WMnMPRAGE space) in order to compare with the 3T post‐treatment WMnMPRAGE scan showing the ablation. These standard initial target coordinates were then linearly transformed into each patient's 3T post‐treatment WMnMPRAGE space. The probabilistic target was then nonlinearly transformed into each patient's 3T post‐treatment WMnMPRAGE space using the reverse transformation generated from nonlinearly warping each patient's pretreatment WMnMPRAGE to generate the group template with ANTs. For each patient, we then computed the fraction of either the probabilistic target region or a sphere of the same volume centered on the standard initial target region that was covered by the ablation core. These overlap fractions were plotted against 1‐month clinical outcome across subjects to assess whether the probabilistic target region or the standard initial target was better at predicting outcome at 1 month.

### 2.9 Statistical methods

We evaluated how well these image‐derived metrics correlated with clinical outcome in the dominant hand 1 month after treatment according to the CRST A + B (Stacy et al., 2007). A Wilcoxon signed‐rank test with continuity correction was used to evaluate whether the treatment led to a significant improvement in the clinical outcome of patients 1 month after treatment as measured by their CRST A + B scores. Pearson correlations between image‐derived metrics and the patients' 1‐month clinical outcomes were compared to better understand why some procedures were more effective at reducing tremor than others and to evaluate new targeting strategies.

## 3 RESULTS

### 3.1 WMnMPRAGE allows manual segmentation of the Vim in individual ET patients

We found that WMnMPRAGE images (acquired at 7T for all patients except Patient C, for whom these images were acquired at 3T) provided sufficient contrast to allow manual segmentation of individual thalamic nuclei, including VLp, the ventral half of which was operationally defined as the Vim (Figure 1). As outlined by Tourdias et al. (2014), VLp was identified in preoperative WMnMPRAGE images by differences in signal intensity between itself and adjacent structures and by thin bands of hypointensity likely corresponding to myelin‐rich lamellae between thalamic nuclei. Medially, VLp is adjacent to the more hyperintense MD nucleus. In many cases, this boundary between VLp and MD was demarcated by a hypointense band that likely corresponds to the internal medullary lamina. Laterally and ventrally, VLp is bounded by strongly hypointense white matter bundles, the internal capsule and thalamic fasciculus, respectively. On its anterior border, VLp is bounded by the more hypointense VA and VLa nuclei. The posterior boundary of VLp is difficult to discern, but the adjacent VPL nucleus is somewhat more hypointense than VLp and ends close to the anterior tip of the pulvinar. In comparison to 3T WMnMPRAGE images, 7T WMnMPRAGE images showed improved thalamic contrast and signal, but worse global signal homogeneity. This made thalamic structures easier to segment at 7T, but the loss of signal in the posterior brain and distortion near the sinuses made the 7T images less suitable for intersubject registration and template creation. Overall, we found that manual segmentation of thalamic structures was possible with WMnMPRAGE images.

### 3.2 THOMAS, an automated segmentation method, accurately identifies the Vim in individual ET patients

**FIGURE 4:**
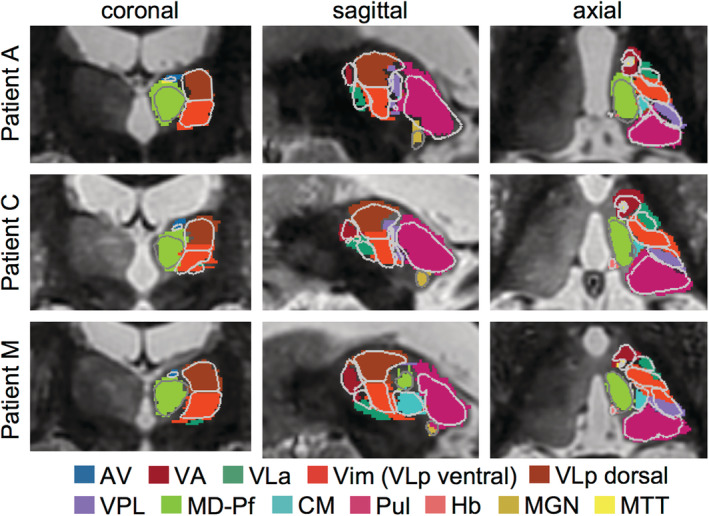
Agreement between manual and THOMAS automated segmentations of the Vim in WMnMPRAGE images. Overlay of manual (filled nuclei) and THOMAS (gray outlines) segmentations in three cases with the best (Patient A, Dice = 0.90), average (Patient C, Dice = 0.74), and worst (Patient M, Dice = 0.55) Dice values for the Vim. Group mean Vim Dice = 0.67 ± 0.07 (see Table 2). Coronal, axial, and sagittal slices are shown centered on the Vim. This analysis used 7T WMnMPRAGE images for all patients, except Patient C, for whom a 3T WMnMPRAGE image was used due to a lack of a 7T WMnMPRAGE image. Thalamic nuclei abbreviations defined in Figure 1

Manual segmentation of the Vim is time intensive and susceptible to subjective bias. To provide a fast, automated, and unbiased method of segmenting the Vim, we examined the accuracy of THOMAS (performed with 7T WMnMPRAGE images for all patients except Patient C, for whom 3T WMnMPRAGE images were used) in detecting the Vim in individual ET patients in comparison to their manual segmentations created from the same images. The degrees of overlap, quantified by Dice and VSI (Table 3), between the THOMAS and manually segmented Vims were mean Dice = 0.67 ± 0.07 and mean VSI = 0.80 ± 0.06, indicating reasonable agreement between THOMAS and manual segmentations (Taha & Hanbury, 2015; Zijdenbos, Dawant, Margolin, & Palmer, 1994; Zou et al., 2004). Figure 4 shows the manual segmentation (filled, colored nuclei) with the THOMAS segmentation overlaid (gray outlines) for three cases with the best (Patient A, Dice = 0.90), average (Patient C, Dice = 0.74), and worst (Patient M, Dice = 0.55) Dice values for the Vim.

**TABLE 3:** THOMAS accurately identifies manually segmented Vim in individual ET patients

Thalamic nucleus	Dice	VSI
AV	0.63 ± 0.17	0.71 ± 0.17
VA	0.60 ± 0.09	0.93 ± 0.03
VLa	0.51 ± 0.10	0.71 ± 0.16
VLp overall	0.76 ± 0.04	0.88 ± 0.05
VLp dorsal	0.79 ± 0.05	0.93 ± 0.06
Vim (VLp ventral)	0.67 ± 0.07	0.80 ± 0.06
VPL	0.58 ± 0.12	0.88 ± 0.09
MD‐Pf	0.82 ± 0.03	0.93 ± 0.04
CM	0.62 ± 0.17	0.90 ± 0.08
Pulvinar	0.83 ± 0.03	0.94 ± 0.04
Habenula	0.72 ± 0.10	0.90 ± 0.07
MGN	0.60 ± 0.09	0.80 ± 0.13
LGN	0.56 ± 0.16	0.81 ± 0.14
MTT	0.60 ± 0.16	0.87 ± 0.10

*Note:* Group means and standard deviations of overlap, as quantified by Dice and VSI, were computed between THOMAS and manual segmentations of the Vim and other thalamic structures using 7T WMnMPRAGE images (except for Patient C, for whom 3T WMnMPRAGE images were used). Thalamic nuclei abbreviations defined in Figure 1.

### 3.3 WMnMPRAGE reveals the core and penumbra of the FUS ablation

**FIGURE 5:**
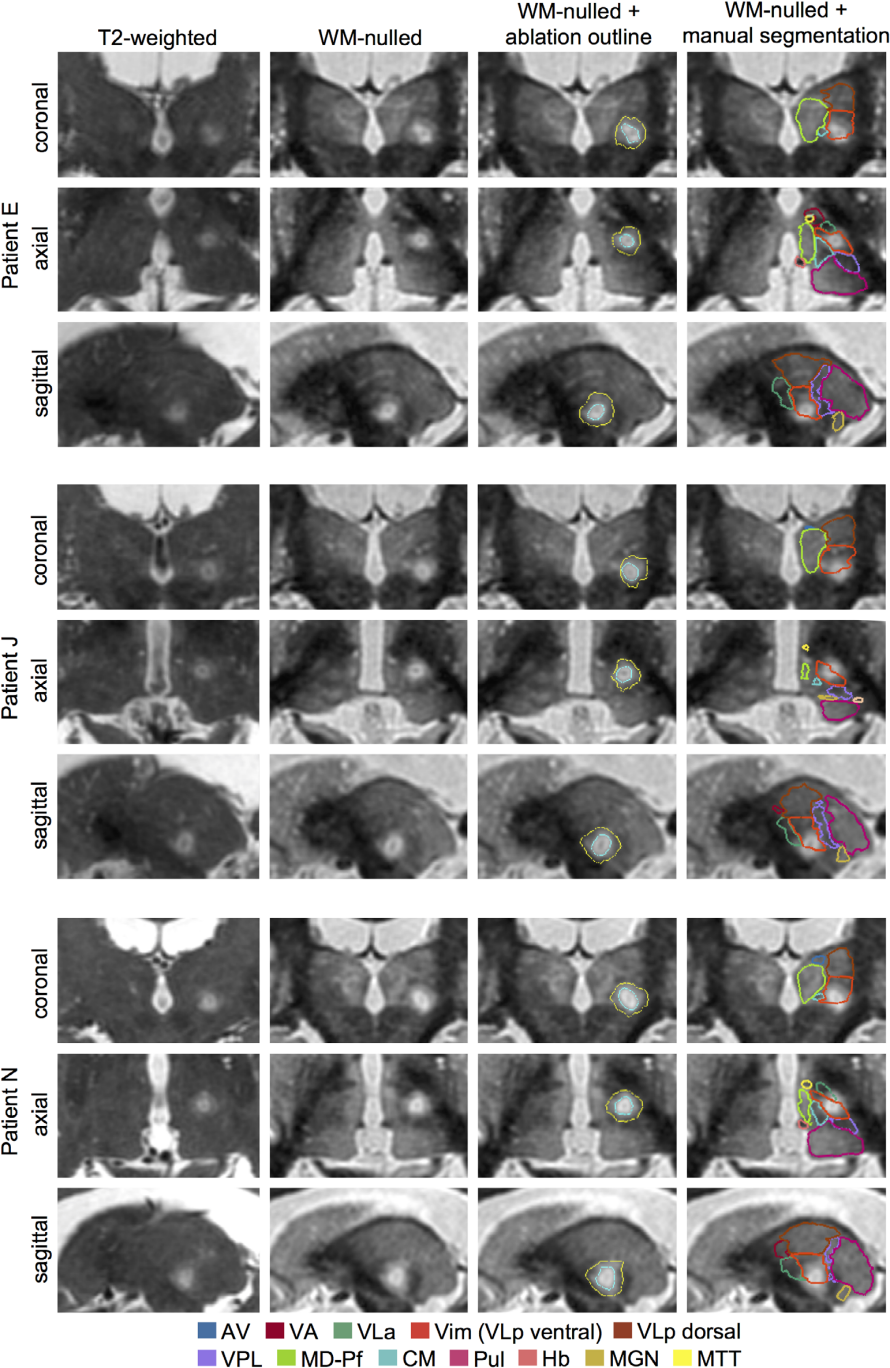
Comparison of immediate post‐treatment 3T WMnMPRAGE images and standard T2‐weighted images in three representative patients. Coronal, axial, and sagittal slices are shown centered on the FUS ablation. Note the improved visibility of the ablation in the WMnMPRAGE images compared to the T2‐weighted images, allowing clearer delineation of an outer penumbra (yellow outline), more hyperintense core (cyan outline), and, in many cases, a hypointense region within the core. For all patients, the ablations were located within and around the Vim (orange outline) defined by manual segmentation in the WMnMPRAGE images. Thalamic nuclei abbreviations defined in Figure 1

The WMnMPRAGE images showed the core and penumbra of the FUS ablations with good clarity (Figure 5). The post‐treatment 3T WMnMPRAGE images showed a hyperintense region in the FUS ablation zone (Figure 5). This zone exhibited a small focal hyperintense core surrounded by a larger and more diffusely hyperintense periphery. The peripheral zone, which we term the penumbra, typically appeared smoother in texture compared to the normal thalamus. In many subjects, an even smaller hypointensity appeared centrally within the core, but this was hard to segment accurately due to its size (typically 3–4 voxels or 0.75–1 mm^3^). For this reason, only the core and penumbra were segmented with a semi‐automated segmentation algorithm.

### 3.4 Comparison of the ablation with the Vim boundaries for individual patients and its relationship with clinical outcome

We examined where the ablation was located relative to the manually segmented Vim within each patient (Figure 5). We assessed the overlap between each patient's ablation and manual segmentation of the Vim that had been drawn independently in the preoperative WMnMPRAGE image. We found that all patients' ablations were within their manually defined Vim and, in many cases, partially overlapping with the ventral boundary of the Vim. In most cases, the penumbra additionally encroached into adjacent nuclei.

We next investigated whether any aspects of the ablation and its location relative to the Vim would correlate with clinical outcome as measured by CRST A + B scores. We found no significant correlations across patients between 1‐month clinical outcome and any of the following measures in group normalized space: the volume of the ablation core (*r* = −.29, *p* = .32), the volume of the whole ablation (core and penumbra combined) (*r* = −.26, *p* = .36), the fraction of the Vim overlapped by the ablation core (*r* = −.35, *p* = .23), the fraction of the Vim overlapped by the whole ablation (*r* = −.45, *p* = .10), the distance between the center of mass of the ablation core to the center of mass of the Vim (*r* = .42, *p* = .13), and the distance between the center of mass of the ablation core to the standard initial target (*r* = −.21, *p* = .48).

### 3.5 Patient ablations intersect in a probabilistic region within the Vim that correlates with clinical outcome

**FIGURE 6:**
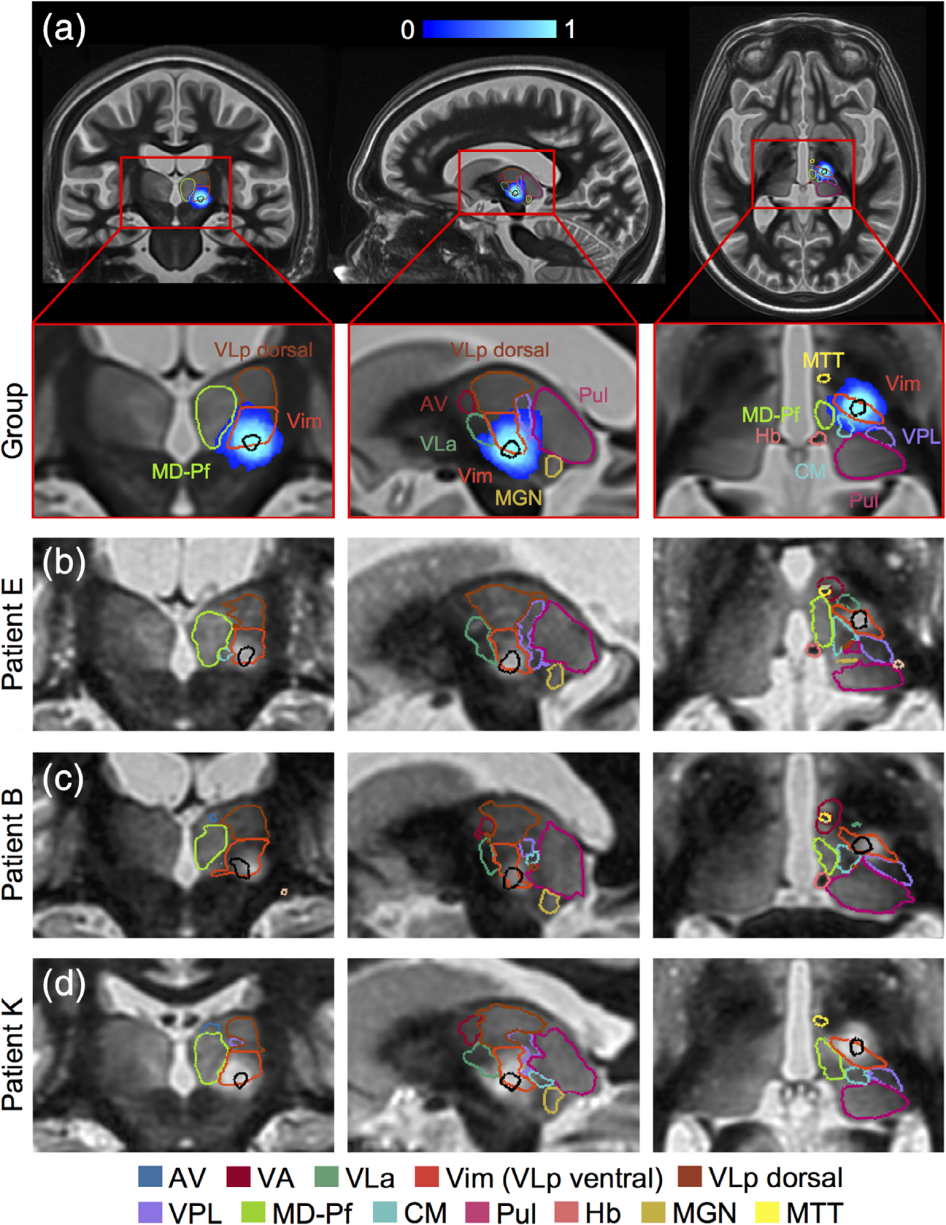
A probabilistic initial target region identified within the Vim. A probabilistic target region (black outline) was defined as the region of 100% overlap of patient whole ablations (blue heatmap), eroded to the mean volume of patient ablation cores. Coronal, axial, and sagittal slices are shown centered on the probabilistic target region in (a) group normalized space with the group thalamic segmentation overlaid and in (b–d) patient native space, by nonlinearly warping the target region from group normalized to patient native space, for three representative patients with their respective manual thalamic segmentations overlaid. Thalamic nuclei abbreviations defined in Figure 1

Having found no significant relationships between clinical outcome and ablation location or size at the individual patient level, we asked whether a probabilistic region based on the intersection of whole ablation regions across the patient cohort would reveal an efficacious target region. Since all subjects consistently experienced at least some clinical improvement immediately following treatment, we hypothesized that the region of the thalamus that was commonly ablated by FUS across all subjects as seen in the immediate post‐treatment imaging would correspond to the optimal treatment target. We found that this probabilistic target region was centered just inside the inferior border of the Vim and centrally situated with respect to the medial‐lateral and anterior–posterior extents of the Vim in the group normalized space (Figure 6a). We then examined whether this probabilistic target region was associated with improved clinical outcome. We found that the greater the fraction of this probabilistic region covered by each patient's ablation core, the lower the patient's tremor score at 1 month post‐surgery; this linear correlation was now statistically significant (*r* = −.58, *p* = .03), which was not the case for any other target or proximity metric that we examined.

### 3.6 The probabilistic target region is a better predictor for clinical outcome than the standard initial target region

We next examined how our probabilistic target region compared to the standard, stereotactic target in predicting clinical outcome. We conducted this analysis in each patient's native space, simulating a new patient case, by nonlinearly transforming the probabilistic target to each patient's native space. As in group template space, the probabilistic target region was centered just inside the inferior border of the Vim in each patient (Figure 6b–d). The target region was also generally centered medial‐laterally and anterior–posteriorly across patients (Figure 6b), with some small individual variation in some cases (Figure 6c,d).

**FIGURE 7:**
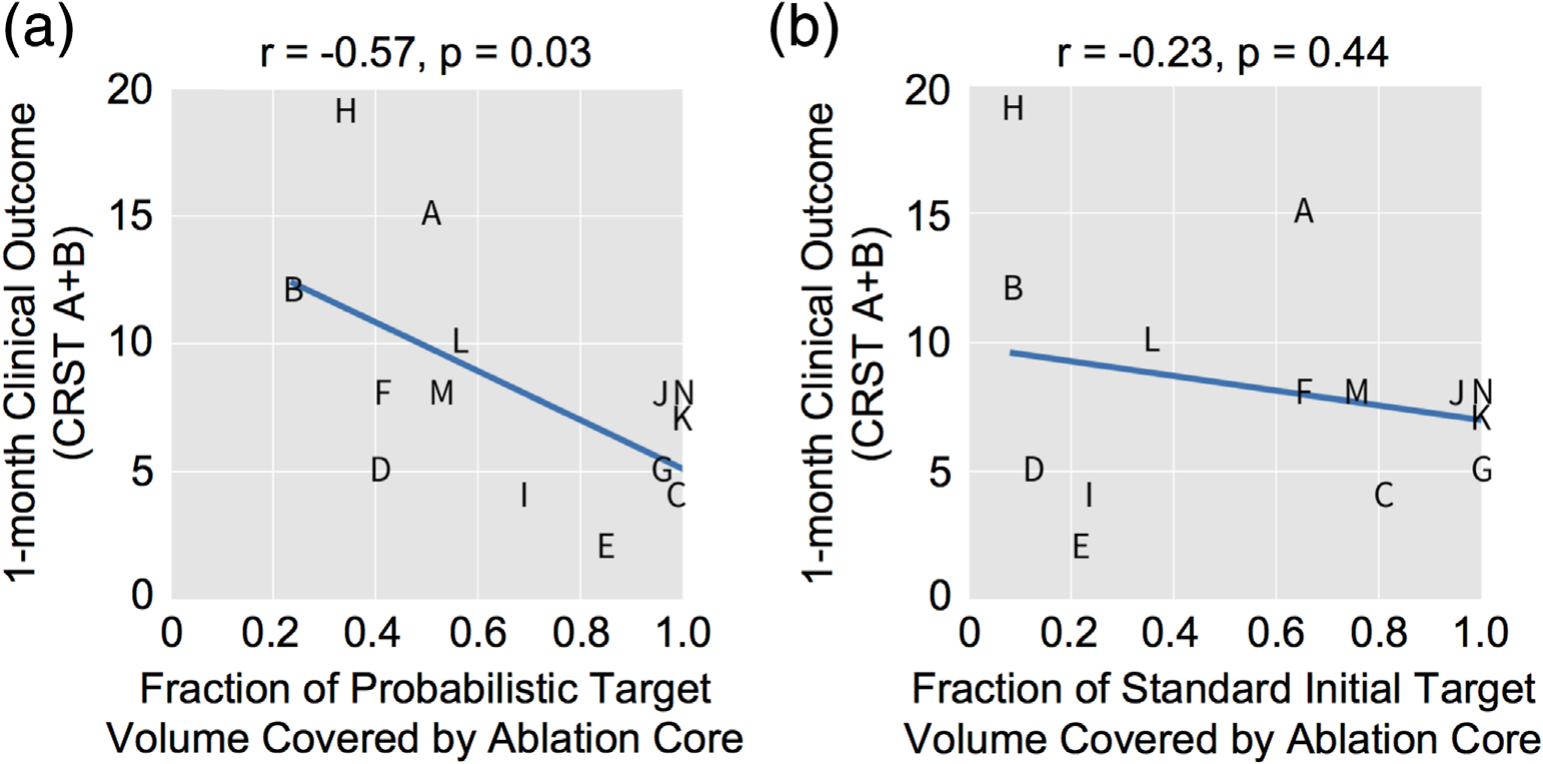
The probabilistic target region identified within the Vim is significantly associated with 1‐month clinical outcome, whereas the standard stereotactically defined target region is not. (a) There is a significant correlation between the fraction of the probabilistic target region covered by the patient's ablation core and 1‐month clinical outcome (CRST A + B), indicating that the greater the overlap between ablation core and probabilistic target region, the better the 1‐month clinical outcome. (b) No significant correlation was observed between the fraction of the standard stereotactic target region covered by the patient's ablation core and 1‐month clinical outcome (CRST A + B)

Similar to that seen in group template space, when analyzed in native space, we found a significant correlation between lower tremor score at 1 month and the fraction of the probabilistic target covered by the patient's ablation core (*r* = −.57, *p* = .03) (Figure 7a). In contrast, the same analysis using the standard initial target region showed no significant correlation between tremor score at 1 month and the fraction of the standard initial target covered by the patient's ablation core (*r* = −.23, *p* = .44) (Figure 7b). This suggests that our probabilistic target region is more clinically efficacious than the standard initial target region.

## 4 DISCUSSION

In this study, we made use of an MR pulse sequence (WMnMPRAGE) with high gray matter‐white matter and intrathalamic contrast, operating at both 3T and 7T, in the pursuit of several important goals: (a) to directly visualize and segment the Vim (defined here as the ventral half of VLp) both manually and with the THOMAS automated thalamic segmentation method in 14 individual ET patients; (b) to identify relevant FUS ablation zones; and (c) to retrospectively identify an upper extremity tremor associated location within each patient's Vim, to be used as an improved initial target for MRgFUS treatment of ET. As we have seen previously in healthy subjects and multiple sclerosis patients (Planche et al., 2019; Tourdias et al., 2014), WMnMPRAGE images showed sufficient contrast in the thalamus to allow manual and THOMAS segmentation of individual thalamic nuclei, including the Vim, in individual ET patients. WMnMPRAGE also shows a clear depiction of the FUS ablation, identifying the core and penumbra zones. The patient ablation cores, which were placed independently using the standard stereotactic targeting method during the MRgFUS procedure, were all located within the Vim segmentation boundaries. In searching for a metric of clinical efficacy and targeting accuracy, we found that none of the individual patient‐level ablation features, such as ablation location or size, correlated with clinical outcome. However, we found that the amount of overlap between a group‐based probabilistic target region, which was nonlinearly warped into patient native space and found to lie within Vim boundaries for all patients, with the ablation core of each patient correlated significantly with clinical outcome at 1 month. The same analysis using the standard target region showed no significant correlation with clinical outcome at 1 month.

Altogether, we found that WMnMPRAGE is a highly sensitive imaging sequence for detecting the Vim, other thalamic nuclei, and the core and penumbra of a FUS ablation for a given ET patient. We also report an optimized initial target region for ET‐FUS ablation that is more significantly associated with clinical outcome at 1 month than the standard, stereotactic method of initial targeting. Using high quality preoperative and postoperative WMnMPRAGE imaging, state‐of‐the‐art nonlinear registration, segmentation of thalamic nuclei and FUS ablation characteristics, and probabilistic lesion calculations using a cohort of ET patients, we were able to discover and define a location within the Vim that is associated with upper extremity tremor reduction. This is a powerful example of human brain mapping that cannot be achieved in healthy subjects, yet still involves entirely noninvasive imaging and therapeutic modalities.

### 4.1 WMnMPRAGE allows direct visualization of the Vim in individual patients

There are significant patient‐to‐patient variations in brain anatomical features, including AC‐PC characteristics (Anthofer et al., 2014; Brierley & Beck, 1959). For this reason, our ability to accurately identify and segment the Vim in an individual patient should be a significant advantage for preoperative planning for ET‐FUS, as well as other treatments that target the Vim. Currently, most imaging protocols are unable to directly depict the Vim, which has led to the standard use of indirect methods like stereotactic targeting relative to visible anatomical landmarks, such as the ventricular wall, AC, PC, and, more recently, the fornix (King et al., 2017). As we showed previously with healthy subjects and multiple sclerosis patients (Tourdias et al., 2014), we found that WMnMPRAGE images acquired at 3T or 7T provide sufficient contrast to directly visualize and allow manual segmentation of individual thalamic nuclei, including the Vim, in individual ET patients. The accuracy of our method is supported by our finding that the ablation cores of all 14 subjects in our study, placed prospectively using the standard stereotactic targeting method, were centered within the patient's Vim boundaries, as defined independently and retrospectively using our WMnMPRAGE and manual segmentation tools.

Several prior studies have also investigated specialized imaging protocols to improve direct visualization of subcortical structures. FGATIR and WAIR are sequences that, like WMnMPRAGE, null the white matter, providing improved visualization of subcortical structures, including thalamic ventral nuclei like the Vim (Sudhyadhom et al., 2009; Vassal et al., 2012). Vassal et al. (2012) prospectively showed that a 1.5T WAIR sequence could be used to manually segment and successfully target the Vim using DBS with good clinical outcome. Morishita et al. (2019) used FGATIR to identify the Vim in individual patients for use in modeling the dentato‐rubro‐thalamic tract with DTI and retrospectively associating this tract with effective DBS contact location. While we have not formally compared WMnMPRAGE to FGATIR or WAIR, our WMnMPRAGE sequence is different from these other MPRAGE‐based sequences in that it uses different sequence parameters and k‐space trajectory, providing robustness to motion, as well as being optimized for SNR efficiency and intrathalamic contrast (Saranathan et al., 2015). Susceptibility‐weighted imaging acquired at 7T (Abosch et al., 2010; Deistung et al., 2013) and proton density mapping (Spiegelmann et al., 2006) have also been demonstrated to allow identification of the Vim in comparison to T1 and T2‐weighted images. Our qualitative comparisons suggest to us that WMnMPRAGE is a highly sensitive sequence that provides improved visualization of the Vim and other thalamic nuclei in comparison to these other sequences. Further study is required, but our results contribute new evidence suggesting that the WMnMPRAGE sequence provides increased ability to identify patient‐specific thalamic anatomy, which can be used to improve clinical FUS treatment of ET.

### 4.2 Automated segmentation of the Vim using THOMAS as a fast, unbiased proxy for manual segmentation

Manual segmentation of the Vim using WMnMPRAGE images, while being the most direct, individualized method of identifying the Vim in each patient, is a manually laborious and time‐consuming process that is susceptible to subjective bias. We found that THOMAS, an automated segmentation method, accurately identifies the Vim with good agreement compared to manual segmentation. THOMAS can thus provide a fast and unbiased alternative for identifying the Vim. This, in conjunction with the probabilistic target region presented here, could assist in more accurate and efficient FUS treatment.

### 4.3 WMnMPRAGE visualizes the core and penumbra of the focused ultrasound ablation

We found that WMnMPRAGE is highly sensitive with regard to delineating the FUS ablation. This allowed us to clearly observe subregions within the ablation: an outer penumbra, inner core, and a hypointense spot within the core. This is similar to what has been observed by others using different MR contrasts. Wintermark et al. (2014) describe three zones (Zones I: necrosis, II: cytotoxic edema, and III: vasogenic edema) using T2‐weighted imaging. Zone I may correspond to our small central hypointense spot, Zone II may correspond to our core, and Zone III may correspond to our penumbra. Although we did not acquire the exact same T2‐weighted imaging as Wintermark et al. (2014), our results suggest that the highly sensitive T1‐based WMnMPRAGE contrast allows for the identification of ablation features with at least as much sensitivity as T2‐weighted imaging. These findings, in combination with the ability to visualize the Vim, strongly support the use of WMnMPRAGE for ET‐FUS pretreatment planning and post‐treatment ablation monitoring.

There have been conflicting results from the literature showing both that ablation volume correlates (Federau et al., 2018; Pineda‐Pardo et al., 2020) or does not correlate (Atkinson et al., 2002; Goodman et al., 1998; Harary et al., 2018; Hariz & Hirabayashi, 1997) with clinical outcome. However, Pineda‐Pardo et al., as well as Boutet et al. (2018), did find that larger lesions were also associated with more adverse effects and note that reliable control over the lesion volume is important. Altogether, these findings underscore the importance of precise, personalized Vim targeting.

### 4.4 A clinically efficacious target region identified within the Vim

While direct visualization of the Vim is critical for individualized targeting, the Vim still spans a relatively large volume that encompasses a somatotopic representation of the whole body. A goal in FUS treatment of ET is to identify a “sweet spot” within the Vim that will maximize clinical efficacy and minimize procedure time and the adverse effects of FUS, including reducing the number of iterations of tissue heating needed intraoperatively to identify the optimal treatment location and perhaps eventually allowing FUS to be conducted fully under anesthesia. The probabilistic target region in the present work is a group‐consensus region created retrospectively from 14 patients treated using the standard stereotactic targeting method. Unlike the standard stereotactically derived initial target region, this probabilistic target region was derived in a data‐driven manner and reflects the common location of ablation across all patients. It was created based on the reasoning that all patients felt some relief of tremor symptoms immediately after the operation, and the region common to all patients is potentially the most clinically efficacious location across patients that would be ideal for initial targeting within the Vim. We found that the degree of volumetric overlap of each patient's ablation core with this probabilistic target region showed significant correlation with tremor reduction at 1 month, while overlap with the standard initial target region showed no significant correlation. This striking result suggests that the probabilistic target region is a more clinically reliable initial target site. Nonetheless, we emphasize that a limitation of our study is its retrospective nature. This means that the probabilistic target region that we identified, derived from and tested for clinical efficacy in the same patients, can only be considered a promising candidate target. A prospective randomized clinical trial, with a larger cohort and time points beyond 1 month, would be required to prove that this novel candidate target region is unequivocally beneficial to patient outcome.

The probabilistic target region in group‐normalized space showed that, across patients, the target region is located dorsal to the inferior border of the Vim and situated centrally in the medial‐lateral and anterior–posterior axes. When nonlinearly transformed to patient native space, the probabilistic target region was also centered just inside the inferior border of the Vim in each patient's native space, although with some small variation in the medial‐lateral and anterior–posterior extents. This small individual variation could be due either to some medial‐lateral and anterior–posterior error in manually segmenting the Vim or to idiosyncratic patient‐specific Vim shapes that are not fully compensated by the nonlinear warping method. Alternatively, this could be due to individual differences in the precise somatotopic map in a patient's Vim or the somatotopic location of the tremor. Further study is needed to clarify this question.

Much recent work has investigated personalized Vim targeting through the use of connectivity‐derived definitions of the Vim, primarily based on DTI. These DTI‐based approaches include identifying the Vim based on its known connection with the dentato‐rubro‐thalamic tract (Chazen et al., 2018; Coenen et al., 2017; Fenoy & Schiess, 2017, 2018; King et al., 2017; Low et al., 2019; Miller et al., 2019; Morishita et al., 2019; Sammartino et al., 2016; Sasada et al., 2017) or bounding fiber bundles (Krishna et al., 2019; Ranjan et al., 2019) or by segmenting the thalamus based on connectivity with the cerebral cortex (Akram et al., 2018; Middlebrooks et al., 2018; Pouratian et al., 2011; Tian et al., 2018; Tsolaki, Downes, Speier, Elias, & Pouratian, 2018). There is accumulating evidence that this connectivity‐based targeting provides an individualized initial targeting method associated with good clinical outcome. However, there has also been debate as to which seed regions of interest to use for DTI, technical difficulties associated with DTI for the thalamus, and differences across studies on the precise “sweet spot” within the Vim to target, making the optimal connectivity‐based targeting method still an open question (Akram et al., 2019; Middlebrooks et al., 2019). Thus far, the leading definition of the sweet spot is the thalamic region intersecting with the dentato‐rubro‐thalamic tract (Calabrese et al., 2015; Akram et al., 2018; Chazen et al., 2018; Fenoy & Schiess, 2017, 2018; Weidman, Kaplitt, Strybing, & Chazen, 2019; Morishita et al., 2019; Low et al., 2019; Miller et al., 2019; but see Schlaier et al., 2015). Additional reported sweet spots across the literature include thalamic regions connected with M1, SMA, or premotor cortex (Akram et al., 2018; Middlebrooks et al., 2018; Pouratian et al., 2011; Tsolaki et al., 2018), and near the inferior and posterior boundary of the Vim (Al‐Fatly et al., 2019; Atkinson et al., 2002; Boutet et al., 2018).

In comparison to these connectivity‐based methods for targeting Vim, we believe that our approach, based on high‐resolution, high‐contrast WMnMPRAGE structural imaging that permits direct, patient‐specific segmentation of the Vim and nonlinear warping of the probabilistic target region to patient native space based on whole brain anatomy, is a more direct method for defining the Vim. Future studies are needed to directly compare our probabilistic target region with alternative methods and sweet spots reported in the literature.

### 4.5 Recommendations for Vim targeting to treat essential tremor

Based on our retrospective analysis of treatment efficacy in 14 patients, we believe that the clinically efficacious, group‐normalized probabilistic target region reported here is an improved initial target region for ET‐FUS planning compared to the standard, stereotactic method used for MRgFUS and DBS surgeries. Although the present study has been conducted in the context of focused ultrasound therapy for ET, our probabilistic region may also be an improved target for DBS electrode placement for the treatment of ET. We recommend applying the probabilistic target region (available at github.com/thalamicseg/et-vim; Su et al., 2020) into the patient's native space (e.g., preoperative WMnMPRAGE image), specifically using a nonlinear warp. We believe the nonlinear warp is critical to perform because it accounts not only for the patient's own head size and thalamic anatomy, but also for nonlinear differences in brain anatomy compared to the group template brain. The nonlinear warp means that it is not possible for us to define our probabilistic target using Talairach coordinates (which would imply that that a linear transformation, like a stereotactic approach, should not be used to apply this probabilistic target to an incoming patient's native space). Specifically, our recommended steps are to nonlinearly register the patient's pretreatment WMnMPRAGE image to the study‐specific template and apply the inverse warp to transfer the probabilistic target region back to the patient's native space. The result is a patient‐specific target derived from in vivo thalamic imaging. This should ideally be done using the patient's preoperative 3T or 7T MRI WMnMPRAGE image, which has high subcortical structural contrast with established capabilities for thalamic segmentation and registration. The use of more conventional T1‐weighted (CSF‐nulled) MPRAGE imaging for this purpose is expected to be inferior, but remains to be investigated.

We also recommend the use of a preoperative WMnMPRAGE to allow identification of the patient's own Vim, either by manual segmentation or application of THOMAS. While this is not strictly necessary if utilizing the above‐stated steps for nonlinearly transforming the probabilistic target region to patient space, a direct visualization of the Vim may provide a valuable check that the target region is within the patient's Vim. Indeed, our data indicates that the identification of patient‐specific Vim and targeting of the center of its inferior border could provide a reasonable approximation of the optimal target for that patient. Furthermore, we recommend the use of postoperative WMnMPRAGE in place of T2‐weighted scans. This latter recommendation is based on the qualitative observation that WMnMPRAGE is the more sensitive MRI contrast for visualizing the core and penumbra of the FUS ablations, allowing the effects of tissue heating to be more easily and precisely monitored over time following ablation.

### 4.6 Technical limitations

One major limitation of our study is that it was a retrospective study in which our probabilistic target region was created from and tested for clinical efficacy in the same patients. An important future test, which was beyond the scope of the present study, would be to assess our probabilistic target region's ability to predict clinical efficacy in an independent set of patients. An alternative future study design might be a prospective randomized clinical trial using two groups of patients with initial targeting performed with our method versus the standard, stereotactic method. Additionally, our study was limited in terms of patient numbers as well as imaging and clinical timepoints. In this study, we examined 14 subjects using immediate post‐FUS imaging and clinical outcomes at 1 month. We were limited to immediate post‐treatment WMnMPRAGE imaging by our study protocol; imaging at later timepoints (i.e., when zone sizes are smaller) would provide more information about the temporal evolution of the lesion and metrics derived from later timepoint imaging may correlate better with outcome. The 1‐month clinical timepoint was chosen based on the availability of a complete set of clinical outcome measurements for all 14 patients in our study. In addition, the 1‐month timepoint is an intermediate timepoint that is sufficiently close in time to the MRgFUS procedure to show strong therapeutic effects (the therapeutic effect has been observed to decrease over time in some patients), but sufficiently far in time from the procedure that edema and adverse events are reduced (Chang et al., 2015; Harary et al., 2018; Jung et al., 2015; Wintermark et al., 2014). Evaluation with clinical outcomes at later timepoints will be critical for assessing the ability of our probabilistic target region to predict longer term therapeutic effects. As MRgFUS becomes more widespread, studies with larger numbers of patients and examining farther timepoints from surgery are becoming more common (Halpern et al., 2019). Future work would benefit from a larger patient cohort and from the correlation of image‐derived features with clinical outcome at multiple timepoints post‐treatment.

## 5 CONCLUSIONS

Thalamic imaging using WMnMPRAGE, acquired before and after MRgFUS treatment of ET, has allowed us to directly visualize and manually segment patient‐specific thalamic anatomy, including the Vim nucleus, as well as FUS ablation characteristics. We report high accuracy in an automated patient‐specific segmentation of the Vim using THOMAS, referenced against manual segmentation in the same subjects. We also report a group‐based probabilistic target region within the Vim that is better associated with clinical efficacy than the standard, stereotactically defined target, identifying a novel upper extremity tremor center and an improved initial FUS target. These new tools and findings may better equip MRgFUS to deliver effective patient‐specific therapy. The group template and the probabilistic target regions are available online (github.com/thalamicseg/et-vim).

## CONFLICT OF INTERESTS

P. G. and C. H. H. receive research funding from INSIGHTEC. C. H. H. receives consulting fees from Boston Scientific, Ad‐Tech, and Medtronic. B. K. R. receives research funding from GE Healthcare. The other authors have no other conflicts to declare.

## DATA AVAILABILITY STATEMENT

The probabilistic target region and group average brain template are openly available in GitHub at https://github.com/thalamicseg/et-vim. THOMAS, the automated thalamic segmentation algorithm, was made openly available by Su et al. (2019) in GitHub at https://github.com/sujason/thomas.
